# A Domain-General Monitoring Account of Bilingual Language Control in Recognition: The Role of Language Dominance and Bilingual Experience

**DOI:** 10.3389/fpsyg.2022.854898

**Published:** 2022-04-12

**Authors:** Ruilin Wu, Esli Struys

**Affiliations:** ^1^Centre for Linguistics, Department of Linguistics and Literary Studies, Vrije Universiteit Brussel, Brussels, Belgium; ^2^Centre for Neurosciences, Vrije Universiteit Brussel, Brussels, Belgium

**Keywords:** minority language, language dominance, cognitive control, flanker task, word recognition

## Abstract

The ability of bilingual individuals to manage two competing languages is assumed to rely on both domain-specific language control and domain-general control mechanisms. However, previous studies have reported mixed findings about the extent and nature of cross-domain generality. The present study examined the role of language dominance, along with bilingual language experience, in the relationship between word recognition and domain-general cognitive control. Two single-language lexical decision tasks (one in L1 and another in L2) and a domain-general flanker task were administered to bilinguals who live in the sociolinguistic context of a minority and a majority language, namely, Uyghur (L1) and Chinese (L2), respectively. The results showed a diversity in language dominance patterns with better performance in L2 than L1 in the recognition modality, even for participants who self-identified as globally being dominant in L1. This finding reflected all bilinguals’ self-evaluation that their preferred language for reading was L2, suggesting that language dominance is dynamic, depending on what language modality is measured. Furthermore, it was found that an earlier onset age of L2 acquisition (but not recent exposure) and a higher across-modality dominance in L2 were related to faster L2 word recognition. When self-reported language dominance was operationalised as a grouping variable, it was further found that both across-modality L1- and L2-dominant bilingual participants demonstrated a significant relationship between L2 word recognition and domain-general monitoring control, while only L1-dominant bilinguals additionally tapped into inhibitory control, indexed by the flanker effect during L2 word recognition. These findings suggest that language dominance has an impact on the extent and nature of the overlap in control mechanisms across specific linguistic and domain-general cognitive domains and add evidence to a domain-general monitoring account of bilingual word recognition.

## Introduction

The ability of the bilingual mind to restrict lexical access to appropriate lexical representations in the word recognition process has aroused considerable attention from researchers. Numerous recent studies have shaped the account that in the domain of language recognition, irrespective of a single or dual-language context, lexical stimuli non-selectively activate lexical representations in the target language as well as competitors from the non-target language (Linck et al., 2008; van Assche et al., 2009; Wu and Thierry, 2010; Zhou et al., 2010; Moon and Jiang, 2012; Nakayama et al., 2012; Miwa et al., 2014; Hoversten et al., 2015; Gangopadhyay et al., 2019). Evidence for non-selective co-activation has been found in languages with the same script, such as Dutch–English (de Groot et al., 2000; Dijkstra et al., 2000; Lemhofer and Dijkstra, 2004; van Heuven et al., 2008; van Assche et al., 2009) and Spanish–English (Macizo et al., 2010; Hoversten et al., 2015; Pu et al., 2019). However, it has also been found in bilingual individuals (henceforth, bilinguals) who speak two languages with distinct scripts, such as Chinese–English (Wu and Thierry, 2010; Zhou et al., 2010), Japanese–English (Nakayama et al., 2012; Miwa et al., 2014) and Korean–English (Moon and Jiang, 2012). For instance, Wu and Thierry (2010) tested Chinese–English bilinguals in a single L2-English context in which English words were presented in pairs and participants had to decide on their semantic relatedness, but bilingual participants were unaware that some semantic-unrelated word pairs had an implicit feature, such as a sound repetition in the L1-Chinese translation (e.g., the word pair ‘classic–surprise’ translated into Chinese with a sound repetition of ‘jing’: ‘jingdian–jingya’). Based on the analysis of the neuroimaging technique of event-related potentials (ERP), it was found that the implicit sound repetition in the Chinese translations induced a positive priming (facilitating) effect in judging the semantic relatedness in English. This result suggests that for two languages with distinct scripts, processes underlying L2 word recognition also imply the activation of L1 lexical items.

Even though these studies have shown that language non-selective access exists in the bilingual recognition process, it is still unclear what type of bilingual language control is involved in addressing the competition of the co-activated languages and in selecting the appropriate language. Regarding the underlying mechanism of bilingual language control for language selection, inhibitory control may serve an important role during the processes of bilingual language production and recognition. In the inhibitory control (IC) model proposed by Green (1998), the selection of the intended language in bilingual speech production is realised through the language task schemas to exert a top-down (domain-general) inhibitory control over the interference of the co-activated but competing lexical representations from the unintended language. Moreover, Green and Abutalebi (2013) proposed that bilingual speakers’ demands in cognitive control may be adaptive to different interactional language contexts. For instance, more inhibitory control may be recruited when bilinguals are exposed to single- and dual-language contexts than to a dense code-switching context, because the latter context featuring a high frequency of language switching may constitute a more cooperative than conflictual relationship between the two languages, compared to the former two interactional language contexts.

Concerning bilingual visual word recognition, the theoretical model of bilingual interactive activation (BIA; Grainger and Dijkstra, 1992; van Heuven et al., 1998) suggests that the lexical input activates the two competing languages, and the lexical items in the non-relevant language are suppressed by receiving domain-general inhibitory control *via* language nodes (indicating language membership); in turn, the relevant language remains highly activated due to lexical features maximally corresponding to the input stimuli and is then selected. Nevertheless, the succeeding BIA+ (Dijkstra and van Heuven, 2002) and multilink models (Dijkstra et al., 2019) remove the role of inhibitory control and draw a distinction between the encapsulated word identification system and the task/decision system. Paap et al. (2019) have further proposed that active maintenance and selection of the relevant language can be sufficiently realised through an inhibitory mechanism that is part of the language processing system itself rather than through the recruitment of (domain-general) cognitive inhibitory control.

Regarding whether domain-general inhibition plays a central role in bilingual language process, a recent study by Bialystok and Craik (2022) provides an alternative account based on attentional control. It is argued that attentional control, namely, abilities to guide attention to the target stimulus, may be a better account for the underlying mechanism of bilinguals than inhibitory control which emphasises abilities to suppress the non-target distractor. It further suggests that no matter which domain-general control is involved, bilinguals only recruit domain-general control when the task demands an excessive amount of control abilities.

### Language Control and Domain-General Control in Bilingual Language Recognition

Concerning the involvement of domain-general control mechanisms in bilingual language recognition, a growing number of studies (Blumenfeld and Marian, 2011; Blumenfeld et al., 2016; Freeman et al., 2017) have started to investigate the direct relationship between domain-specific (linguistic) control and domain-general control. Specifically, researchers have adopted the correlational approach, in which bilinguals’ performance on a word recognition task (as an indication of bilingual language control) is compared to their performance on a non-verbal task (as an indication of domain-general control). For instance, Freeman et al. (2017) measured Spanish–English bilinguals’ language recognition control with a priming version of a single-language (English) lexical decision task where an English auditory prime preceded the visual presentation of an English stimulus (word or a non-word), and participants were required to decide whether the stimulus was a real English word or not. When the auditory prime was a cognate (a word in English and Spanish with similarity in form [spelling and sound] and meaning), the non-target Spanish pronunciation was supposed to be highly activated. Therefore, if the succeeding English non-word stimulus overlapped with this cognate prime in the phonological form (e.g., cognate prime ‘stable’ [‘estable’ in Spanish]; non-word stimulus: ‘esteriors’), substantial cross-language competition might be elicited. It was found that a smaller Stroop effect (better cognitive control) was correlated with reduced cross-language interference (indexed by the difference between a non-word with phonological overlap with the preceding cognate prime and a non-word with no phonological overlap with the preceding cognate prime), elicited by phonological co-activation due to the presence of cognates.

Even if the non-target language is not being manipulated, as was the case in the study by Freeman et al. (2017) through manipulation of the cognate status of the target language, a domain-general contribution to bilingual language recognition in a single-language lexical decision task can be observed. Gangopadhyay et al. (2019) conducted an auditory version of a single-language (English) lexical decision task to measure language recognition control and two non-linguistic tasks (a flanker task as an indication of interference suppression at the stimulus level and a go/no-go task as an indication of response inhibition) to measure inhibitory control. The same set of linguistic and cognitive tasks were administered to bilinguals and monolinguals at two separate time points (i.e., years 1 and 2) with an interval of a year. At both years 1 and 2, better domain-general inhibition in the bilingual participants was associated with more accurate (but not faster) recognition processing of both words and non-words. Moreover, in the longitudinal analyses, it was found that higher overall accuracy (with both words and non-words) on the language task in year 1 may predict better inhibitory control in year 2.

Other studies have used a language switching paradigm to measure language control in the process of visual word recognition (e.g., Struys et al., 2019) and have compared performance on switch trials of these tasks with domain-general control. Using this methodology, Struys et al. (2019) proposed that sustained and proactive monitoring control indexed by overall performance in the Simon task was the driving mechanism underlying bilingual language recognition.

While these studies suggest domain-general cognitive involvement in language control, not all studies and tasks investigating the relationship between domain-specific and domain-general control have consistently found this involvement (for a review, see Calabria et al., 2018). The following section will explore the proposition by Anthony and Blumenfeld (2019) that these contradictory results may stem from unclear distinctions between bilinguals in terms of bilingual profiles. Language dominance is suggested to play a role in the degree of the link between linguistic control and cognitive control.

### The Role of Language Dominance in Cross-Domain Overlap

There is some evidence that bilinguals with high proficiency in an L2 perform more efficiently compared to L1-dominant bilinguals in language control (e.g., Anthony and Blumenfeld, 2019) and domain-general cognitive control (e.g., Tse and Altarriba, 2015). These findings suggest that when L2 proficiency increases, bilinguals more easily obtain access to L2 lexical–semantic representations. This finding can be theoretically explained by the BIA model (Grainger and Dijkstra, 1992; van Heuven et al., 1998). With respect to bilingual language recognition, the BIA model proposes that language recognition is characterised by a bottom-up activation of the interactive network of lexical representations from two languages; therefore, the process of word identification is highly dependent on the resting-level activation or initial strength of lexical activation at rest. The language in which the bilingual is highly proficient possesses a greater initial strength in activation than the less-dominant language, indicating that when L2 proficiency rises, bilingual language recognition in bilinguals with high L2 proficiency may differ from the same process in bilinguals with low L2 proficiency. A series of language recognition studies using the masked translation priming paradigm demonstrated that for bilinguals with high proficiency in L2, the non-target language (L2) translation equivalent of the target language (L1) was found to facilitate lexical identification in the target L1, whereas L1-dominant bilinguals showed no or only a limited priming effect in the L2–L1 direction (Basnight-Brown and Altarriba, 2007; Perea et al., 2008; Dunabeitia et al., 2010; Wang, 2013; Nakayama et al., 2016; but see Lee et al., 2018).

The role of language dominance in bilingual recognition control is also shown in studies using a language comprehension version of the language switching paradigm in which words are visually presented, with a distinction between repeat trials (two consecutive trials in the same language) or switch trials (the prior and succeeding trial in different languages; for a review, see Declerck and Philipp, 2015). It is proposed that if bilingual recognition control recruits inhibitory control, the dominant language may need to be highly inhibited when words are presented in the non-dominant language; it may then require a higher cost to reactivate the dominant language than the non-dominant language when it was previously the non-target language. Some studies (e.g., Litcofsky and van Hell, 2017; Mosca and de Bot, 2017) have shown that language dominance has an impact on the degree or nature of bilingual recognition control in that larger switch costs existed when switching into the L1-dominant language than into the less-dominant L2. Bultena et al. (2015) indicated that the degree of cost when switching into an L2 was related to the level of L2 proficiency. However, other studies have reported symmetrical costs between switching into a strong L1 and weak L2 (Thomas and Allport, 2000; Macizo et al., 2012; Struys et al., 2019). This absence of a language dominance effect may suggest that language dominance plays a limited role in language recognition control.

A moderating role of language dominance on the connection between bilingual language and domain-general cognitive control can be deduced, therefore, from the difference in linguistic performance between proficient or non-proficient bilinguals. However, despite this indirect evidence, few studies have sought direct evidence of the role of language dominance in the cross-domain relationship—that is, the effect of language dominance on the direct correlation between linguistic and non-linguistic performance. More research is needed on the effect of language dominance in this respect.

### Effect of Sociolinguistic Context on Language or Cognitive Control

Another factor that may have an impact on the overlap between domain-specific and domain-general control mechanisms is the sociolinguistic context to which bilingual individuals are exposed. According to the adaptive control hypothesis (Green and Abutalebi, 2013), bilingual language control mechanisms adapt to various patterns of language use, which may be related to the sociolinguistic context of bilingual interaction. Bosma and Blom (2019) found that bilinguals in a sociolinguistic context with a minority (L1) and majority (L2) language pair can experience considerable adaptability in language control. A majority language has a predominant status in a wide range of interactional language contexts, whereas a minority language typically has a less official status, is restricted to a few interactional contexts (mostly at home in family settings) and is exclusively spoken by indigenous people or immigrants in that region. Because of these differences in status, lexical or grammatical insertions from the majority language into the minority language occur much more frequently than from the minority into the majority language (Couto and Gullberg, 2019). This linguistic phenomenon has an effect on the recruitment of cognitive control networks. Regarding the modality of language production, Bosma and Blom demonstrated that when a conversation was initiated in the L2 majority language (i.e., limited switching into the minority language at the sociolinguistic level), inhibitory control was required to maintain the separation of two languages; however, in the other language direction (speaking an L1 minority language initially where the mixing of two languages is allowed and lexical representations in either language can freely be selected in the production stage), no inhibitory control was involved. The extent to which this minority/majority language sociolinguistic effect on cognitive control can be found in the modality of language recognition still needs further exploration.

Tao et al. (2017) provided further evidence for the effect of sociolinguistic environment on domain-general control in a study of Dai (a minority language spoken by ethnic Dai)–Chinese bilinguals. A composite task, comprising an adapted version of a Simon and Stroop task, was adopted to measure attentional control (attending to the interference at the stimulus level when the non-target picture was not semantically related to the target word) and inhibitory control (suppressing the interference at the response level when the position of the non-target stimulus was incongruent with the response key). By considering the effect of language proficiency, the study found that in the L1-minority language block, the highly proficient bilinguals performed better than the non-proficient bilinguals in sustainable attentional control to monitor stimulus-level interference, while in the L2-majority language block, highly proficient bilinguals performed better in inhibitory control than non-proficient bilinguals. These studies offer strong evidence that the sociolinguistic environment may contribute to differences in the domain-general contribution to L1 and L2 word recognition. However, to our knowledge, no study has yet focused on the moderating effect of language dominance on the connection between bilingual recognition and domain-general control in a specific sociolinguistic context, with a dominance shift over time from the minority to the majority language.

### Linguistic Context of the Present Study

The aim of the present study is to explore the role of bilingual language dominance in the interconnection between bilingual word recognition and non-linguistic cognitive control skills in the asymmetrical sociolinguistic context of the Xinjiang Uyghur Autonomous Region (Xinjiang) in China, with Uyghur (minority L1)–Chinese (majority L2) bilinguals as participants. The Uyghur language is the indigenous language of the region and has an official status at all societal levels, from the informal community context to the formal domains of administration, education and social media (Ma, 2009). The Chinese language (or Standard Chinese) is the national language used among all ethnic groups and in all regions in China. In terms of language typology and script systems, the two languages are very distinct. The Uyghur language, as a member of the Altaic language family, is a phonographic language written in a version of the Arabic alphabet, while the Chinese language, belonging to the Sino-Tibetan language family, is a logographic language written in characters composed of strokes.

Diversity exists within the sociolinguistic context in which Uyghur–Chinese bilinguals acquire or use their two languages, particularly regarding their educational background (Ma, 2009; Guo and Gu, 2018). In the formal educational context, Uyghur individuals are able to attend bilingual education schools (i.e., ethnic minority schools or minority/majority joint schools) or Chinese-medium schools. Uyghurs can develop varying degrees of language proficiency, depending on the educational tracks they opt for. For instance, a Uyghur may transfer between distinct education trajectories when they achieve the required academic and language abilities; for example, they may attend bilingual education schools with both Uyghur and Chinese during primary-level instruction and then switch to the track of Chinese-medium schools at the secondary level or vice versa (Ma, 2009). Recent studies on the informal communication context have shown that even though the Uyghur language might play a dominant role in private communication, young Uyghurs with prolonged experience in using Chinese as a language of instruction tend to engage in language switching or mixing with high frequency, particularly when interacting with their siblings and friends (Masut, 2014; Guo and Gu, 2018). In terms of reading, Masut (2014) found that at least 50% of Uyghurs who had attended Chinese-medium schools opted for the exclusive use of Chinese in reading news (either online or through newspapers), magazines and books. Given that sociolinguistic context is critically relevant to individual language proficiency (de Houwer, 2018), it can be inferred that these variations in educational background in the minority–majority sociolinguistic context may result in large intra-group differences in language dominance for Uyghur–Chinese bilinguals.

### Research Questions and Hypotheses of the Current Study

In the present study, the first objective is to explore whether the different degrees of bilingual language dominance in Uyghur–Chinese bilinguals affect visual word recognition. A single-language lexical decision task is employed as this is the most extensively used task for measuring word recognition (Libben and Jarema, 2002). Stimuli in this task are presented only in one language to resemble the activity of real-life reading. Given that BIA model proposes that as language proficiency in one language increases, that language may become highly activated during word recognition, we hypothesise that if language dominance may have an impact on bilinguals’ performance of word recognition in the single-language context, the more dominant the language is for bilinguals, the faster and the more accurate they are in recognising words (or non-words) in that language. However, we expect that the minority–majority bilingual context would constitute an asymmetry in language use for the study participants and that language dominance may be dynamically adapted to this language environment, with continuous exposure to the predominant language changing the relative strength of the two languages (Montrul, 2015; Silva-Corvalán and Treffers-Daller, 2015). Moreover, because language dominance tends to be dynamic in nature, in that distinct language skills or tasks may reflect varying degrees of language dominance (Bahrick et al., 1994; Treffers-Daller, 2015), global (across-modality) language dominance may not fully represent the dynamic feature of language dominance in each language modality. Following this line of thought, it is possible that in the word recognition task, bilinguals who have experienced dominance shift over time from their L1 to their L2 will show faster L2 word recognition. It is also worthwhile examining the relationship between language experience and language control performance as previous studies have demonstrated the effects of short-term language exposure (Bonfieni et al., 2019; Struys et al., 2019) and the age of L2 acquisition (the initial point of long-term L2 exposure) in the language control mechanism (Bonfieni et al., 2019; Sulpizio et al., 2020). We expect factors related to language experience to contribute to language recognition in the L1 or L2 word recognition process.

The second research objective is to investigate the extent to which the variable of language dominance may have an impact on the relationship between domain-specific and domain-general control. In our study, language control is assessed by the lexical decision task, and domain-general cognitive control is examined by a stimulus–stimulus (i.e., the flanker task) compatibility task (Kornblum et al., 1999). The reason for using the stimulus–stimulus cognitive task is that word recognition is a bottom-up process in which bilinguals need to recognise the target word (input stimulus) from the co-activated lexical candidates that share similarity with the target word (interference rising at the stimulus level or word identification). Therefore, analogous to bilingual word recognition, the flanker effect is generated by an overlap between two conflicting dimensions at the stimulus (input) level—that is, the direction of the surrounding arrows (non-target stimulus) and the direction of the central target stimulus. Due to the shared stimulus–stimulus mechanism, we expect the flanker effect to be a proper indication of the inhibitory control mechanisms related to interference suppression that may be involved in word recognition.

Moreover, the flanker task can serve a dual purpose in that it yields measures of inhibitory control and conflict monitoring control, indexed by overall performance (across trial type; Costa et al., 2009; Singh and Mishra, 2013; Struys et al., 2019; Chan et al., 2020), which suits the present study’s examination of both forms of control. Previous studies have revealed a link between auditory comprehension and inhibitory control (e.g., Blumenfeld et al., 2016). Struys et al. (2019) complemented these findings by examining domain generality in visual word recognition, observing that domain-general monitoring was a potential underlying mechanism in bilingual language control. Therefore, it is reasonable to assume that domain-general monitoring control may be relevant for bilingual language recognition.

In the present study, we are interested in the possible contribution of domain-general control mechanisms to various processes related to word recognition in bilinguals, with attention paid to three aspects. The first aspect is the recognition processes of L1 and L2 real words. These measures represent recognition processes by which the identification of the real word can be achieved without a full analysis of the stimulus. The cohort model of word recognition (Marslen-Wilson and Welsh, 1978) proposes that for existing words, the word is recognised once the point of uniqueness is reached because it matches a trace in the mental lexicon and can be retrieved before the full unit-by-unit analysis of the entire word. The second aspect is the underlying mechanisms of the L1 and L2 non-word effect, which reflect the efficiency of lexical rejection, where the identification of the non-existing word requires a full analysis of the stimulus due to the absence of any trace of the non-word that can be retrieved in the mental lexicon. The third aspect is global performance in the word recognition task, which represents overall lexical processing ability while recognising words and rejecting non-words. Based on the theory of BIA model indicating the presence of inhibitory control in the language recognition process, as well as on recent empirical research (e.g., Struys et al., 2019) showing the involvement of monitoring control in language recognition, we expect inhibition and monitoring to be the two underlying mechanisms that suppress interference from possible across- and within-language competitors to the presented words or non-words. That is, we hypothesise that word recognition measured by L1 and L2 word conditions, the non-word effect or the global performance in the language task overlaps with domain-general inhibitory control, indexed by the flanker effect, and with domain-general monitoring control, measured by overall flanker performance.

By taking into consideration the effect of language dominance, we predict that the varying degrees of language dominance elicited in a minority and a majority language context may lead to different patterns of cross-domain generality. Specifically, previous studies (e.g., Costa et al., 2009) have shown that bilinguals with high proficiency in an L2 demonstrated a greater performance in monitoring control compared to monolinguals, especially in a highly demanding context with a comparable probability of encountering a congruent trial and an incongruent trial. It is logical to expect that as the L2 proficiency increases, bilinguals will switch from relying primarily on reactive control to relying primarily on proactive control to address a demanding language competition in which two languages with equal strength and degrees of activation interfere significantly with each other when one language is the target and the other is not. Therefore, we expect bilinguals with higher proficiency (or even dominance) in the L2 to have experienced more demanding language management than those who have maintained unchanging L1 dominance and may consequently have a great dependency on recruiting monitoring control.

The minority/majority sociolinguistic context may generate potential differences in the recruitment of the underlying mechanisms between the L1 and L2. Specifically, the single-language context of the majority L2 may elicit the recruitment of inhibitory control to avoid (sociolinguistically) unwanted intrusions from the L1, while the use of the L1 minority language involves no or limited inhibition of the L2 majority language. Therefore, we expect domain-general inhibitory control to be exploited exclusively in the L2 context. Moreover, considering the effect of language dominance, we further expect that when bilinguals are more dominant in the L1 minority language, a great reliance on inhibitory control may occur to suppress the interference of the dominant L1 in the L2 context.

## Materials and Methods

### Participants

Seventy bilinguals (average age = 19.64 years, *SD* = 1.41; males = 24, females = 46) were recruited as participants. All participants granted informed consent preceding participation in the empirical tasks. The participants spoke Uyghur as their native language and Chinese as their second language and were undergraduate students from a Chinese-medium university in the city of Xi’an in China. All participants were native speakers of the Uyghur language, because they all firstly acquired the language of Uyghur from birth and reported having spoken that language exclusively with their parents; they all acquired Chinese as the second language on average at the age of 6 in the kindergarten or school context. As reviewed in the previous section, the divergent education trajectories of Uyghurs and their different patterns of language use in the informal communicative context may have led to varying degrees of language dominance.

### Language Background LEAP-Q

To further evaluate their language dominance, participants were asked to fill in an adapted version of the Language Experience and Proficiency Questionnaire (LEAP-Q; Marian et al., 2007) to assess their language backgrounds (see Table 1). All 70 participants reported that their three languages (Uyghur, Chinese and English) were acquired sequentially, and all participants acquired the same L1 (Uyghur) and L2 (Chinese). The Uyghur–Chinese bilinguals showed a significant difference [*t* (69) = 7.64, *p* < 0.001] in each language concerning self-evaluated overall language proficiency on an 11-point (from 0 to 10 with 0 included) scale. The average overall proficiency score for L1-Uyghur (*M* = 8.99, *SD* = 1.11) was higher than for L2-Chinese (*M* = 7.87, *SD* = 1.29). However, participants self-evaluated no significant difference [*t* (69) = 1.00, *p* = 0.321] in their preference for language use between using the L1 (*M* = 48.30%, *SD* = 15.40) and L2 (*M* = 45.00%, *SD* = 13.4). Concerning the participants’ self-evaluated scores specifically related to reading preference, L2-Chinese (*M* = 49.50%, *SD* = 14.77) was chosen significantly more frequently [*t*(69) = −2.14, *p* < 0.05] than L1-Uyghur (*M* = 41.71%, *SD* = 17.02) for reading a book, but participants still self-reported that their reading skill for the L1 (*M* = 9.01, *SD* = 1.28) was higher than for the L2 (*M* = 8.03, *SD* = 1.38), *t*(69) = 6.33, *p* < 0.001. This might suggest that in the actual reading activity, participants had the sense that they more frequently used the L2 as a preferred reading language than the L1, but this potential dominance shift over time in reading was not fully reflected in their scores for self-reported reading skill. Furthermore, a closer examination of language proficiency at the individual level of bilinguals showed a pattern of higher proficiency in L2 than L1. This proficiency pattern accounted for 41% of bilingual participants in terms of self-reported overall language proficiency (across skills). Moreover, the same pattern of higher proficiency in L2 than in L1 occurs in 43% of bilingual participants in self-evaluating their writing proficiency, 41% in reading proficiency, 40% in speaking proficiency and 40% in listening proficiency.

**Table 1:** Language background and language dominance information of bilingual participants.

	Uyghur-Chinese bilinguals (*N* = 70)
Age	19.64 (1.41)
Male/Female	24*/*46
IQ	46.51 (5.21)
L1 recent exposure^1^	47.80% (14.48)
L2 recent exposure	40.71% (12.61)
Age of L2 acquisition	6.14 (2.23)
L1-Uyghur proficiency^2^	8.99 (1.11)
L2-Chinese proficiency	7.87 (1.29)
L1-Uyghur use preference (in %)	48.30% (15.40)
L2-Chinese use preference (in %)	45.00% (13.4)
L1-Uyghur strength (composite score)^3^	55.90 (11.90)
L2-Chinese strength (composite score)	50.90 (11.80)
Index of dominance^4^	4.99 (19.20)
Final dominance score (z-score of index of dominance)^5^	0 (1)

1 *Participants self-evaluated their recent language exposure in percentages. The L1 and L2 language exposure did not add up to 100%, because a third language was also reported in the questionnaire*.

2 *Participants self-reported language proficiency range from 0 (low proficient) to 10 (high proficient) on an 11-point Likert-scale for each literacy skill*.

3 *Language strength is the sum of 4 self-reported scores for language proficiency and 3 self-reported scores for language use preference (transformed)*.

4 *Index of dominance was the difference score between L1-Uyghur strength and L2-Chinese strength (subtracting L2-Chinese strength from L1-Uyghur strength)*.

5 *Final z-score of index of dominance ranged from − 2.24 to 2.71 with a mean of 0*.

*Mean values and standard deviations (in parentheses) are presented*.

In the present study, the self-assessment data of language proficiency and language use preference in each language were integrated into one index for each language. Previous studies (Grosjean, 2015; Silva-Corvalán and Treffers-Daller, 2015; Caffarra et al., 2016) have indicated that language dominance is a complex composite that is related to the variation among bilingual individuals in their preferred language across different contexts. Therefore, in addition to language proficiency, language use preference, reflecting an individual’s attitude towards the actual use of each language, can be considered as a meaningful component of language dominance. This method of using language skill and use preference to measure language dominance has been performed in a previous study (Wu and Struys, 2021). Language proficiency in the dominance measure was a self-evaluated score based on a scale from 0 to 10 for each language skill and for each language. Language use preference was self-rated using percentages to indicate what percentage of each language (i.e., L1, L2, and L3) could be used in a specific scenario, such as reading a book, engaging in a conversation and writing a letter, with the sum of the preference percentages for the three languages equalling 100% for each scenario. For example, in the case of reading a book, a respondent might show a preference for spending 50% of the time reading in the L1, 45% in the L2 and the remaining 5% in the L3.

To better integrate the two elements into the dominance measure, the scale needed to be unified, with the preference percentage data transformed into a score on the same scale as language proficiency. The interconnection between the two sets of values is that language preference evaluated in each linguistic context corresponds to the respective skills in each language, such as reading a book, having a conversation (involving listening and speaking) and writing a letter. A three-step procedure for obtaining the composite score for each language was followed. First, the proficiency score for the three languages in terms of a specific literacy skill was added; for example, a bilingual participant’s reading skills evaluated as 9 for the L1, 8 for the L2 and 4 for the L3 yielded a sum score of 21 for reading. Second, the preference percentage relevant to that literacy skill was multiplied with the sum score of that skill; for instance, if the same bilingual reported the preference of reading a book in the L1 as 50%, in the L2 as 45% and in the L3 as 5%, the language preference percentage in relation to reading would be transformed into scores of 10.5 (21*50%) for the L1, 9.45 (21*45%) for the L2 and 1.05 (21*5%) for the L3. Subsequently, following this method, each language had three transformed scores to indicate the participant’s preference in using that language for reading a book, having a conversation and writing a letter. Each language was also assigned four proficiency scores based on two productive and two receptive language skills. In total, a bilingual possessed seven scores for each language. Third, the overall strength of each language was indexed using a composite score obtained by adding all seven scores from proficiency and preference. In the following step to calculate language dominance, the measurement of language dominance for each participant was operationalised according to the two-step method proposed by Treffers-Daller and Korybski (2015). The first step was to represent the index of language dominance by subtracting the composite score of Chinese (L2) from that of Uyghur (L1). Second, a standardised dominance score was obtained by converting the index of dominance into a *z*-score. This final dominance score of the Uyghur–Chinese bilinguals ranged from −2.24 to 2.71, with a mean of 0. In this continuous scale of dominance score, bilinguals’ final score of more approximate to or above +1 means higher L1 dominance, while the final score of more approximate to or below −1 means higher L2 dominance.

### Designed Tasks and Procedure

All 70 participants took part individually in a lexical decision task, a flanker task and a non-verbal Intelligence Quotient (IQ) test sequentially, with short breaks between each task. A block of practice trials was provided for participants to familiarise themselves with the task requirements before the actual execution of each task. The data for all tasks were collected using a Macbook Pro laptop with a 15.4-inch screen. The programming languages HTML 5 and JavaScript were used for the stimulus design and presentation, and the tasks were presented on a Google Chrome browser. The MySQL database was utilised to record data collected for all the tasks.

#### Raven’s Progressive Matrices

Intelligence was tested due to its close relationship with cognitive control (Arffa, 2007). IQ was evaluated using the standard version of Raven’s Progressive Matrices (Raven, 1938), a non-linguistic IQ test that focuses on metacognitive problem-solving and deductive ability and consists of 60 matrices in five blocks (12 matrices for each), which are arranged on an increasing scale of difficulty. The maximum score for the test is 60 points, with one point gained for the correct answer to one matric. The average IQ score for all 70 participants was 46.51 (*SD* = 5.21).

#### Lexical Decision Task

A visual version of a language-specific (single-language) lexical decision task (Meyer and Schvaneveldt, 1971) was adopted in the present study. Two lexical decision tasks were administered (one in L1-Uyghur and another in L2-Chinese), each containing stimuli of words and non-words in the respective language. Given that lexical decision tasks are sensitive to the effects of word frequency and length (de Groot et al., 2002), these factors were taken into account during the selection of real words from the two languages. The Chinese words were selected from the Character Frequency List of Modern Chinese (Da, 2005), while the Uyghur words were high-frequency words from the unpublished raw data of modern Uyghur words collected from Uyghur websites, newspaper and magazine articles (Abliz, 2015, Unpublished data). Using Zipf-frequency scores (van Heuven et al., 2014) as an index of word frequency for the two languages, the Chinese word stimuli (*M* = 5.81, *SD* = 0.35) were comparable [*t* (94) = −1.42, *p* = 0.160] with the Uyghur word stimuli (*M* = 5.94, *SD* = 0.53). In light of the differences between alphabetical and logographical languages, word length across languages was matched as follows: one Uyghur word was composed of four to six letters with one- (e.g., دوست, /dost/, meaning ‘friend’ in English) or two syllables (e.g., كىتاب, /kitab/, meaning ‘book’ in English), while one Chinese word consisted of a single-component character (e.g., 半, /ban/, meaning ‘half’ in English) or a two-radical component character (e.g., 加, /jia/, meaning ‘plus’ in English) with five to seven strokes. Uyghur non-words (four to six letters) were created by randomly using consonants (C) and vowels (V) to form a non-existent composite that violated the syllable structure of CV(C)(C), while Chinese non-words (five to seven strokes) were generated by randomly combining radicals into a non-existent character with the radicals placed incorrectly. Non-words in each language were checked by native speakers of that language to verify that they were non-words. The complete word and non-word stimuli lists are provided in Appendix 1 in Supplementary Data.

The total number of stimuli in each language block was 96 trials, of which 48 were words and 48 non-words. Participants responded to the trials with keyboard presses; they were instructed to press A for real words and L for non-words. Experimental instructions were given in the Chinese language. All the participants were tested with the same order of the Chinese lexical decision task preceding the Uyghur one. All stimuli were presented in random order, and a fixation cross (1,000 ms) was presented prior to a blank interval (250 ms) and the stimulus itself. Each stimulus was presented in black ink on a white background screen and was terminated after a response or lasted for 2000 ms in the absence of a response.

#### Flanker Task

A series of five arrows with exactly the same distance between each arrow was the stimulus in the flanker task (Eriksen and Eriksen, 1974). Participants were instructed to respond to the direction (leftward or rightward) of the central arrow by pressing the left key (A on the keyboard) or right key (L on the keyboard) and to ignore the direction of the distractors or surrounding arrows. In a congruent trial, the direction of the central arrow was the same as that of the flanker arrows, while in a neutral trial, the flanker arrows were straight lines with no direction (e.g., — — → — —). The total number of trials was 126, and all trial types were equally distributed across the entire task (42 congruent, 42 neutral and 42 incongruent). The proportion of each direction was equal (e.g., 21 trials for left pointing and 21 for right pointing in the congruent trial). The stimuli were presented randomly, and each stimulus appeared after a fixation (500 ms), followed by a blank interval (250 ms). The stimulus was on display until participants responded to it or for a maximum of 2,500 ms.

### Analyses

The response times (RTs) of the correct responses and accuracy rates (proportion of correct responses to the total number of trials) from the linguistic and cognitive tasks were included in the analyses. In each task, data trimming was conducted for each participant, with RTs deviating more than 2.5 *SD* from the mean across all correct trials and RTs lower than 300 ms or greater than 1,500 ms excluded (Kaandorp et al., 2017). In the lexical decision task, 3.60% of the data (467 out of 12,986 trials) were eliminated, and 2.29% of the data (199 out of 8,685 trials) in the flanker task were excluded.

## Results

The accuracy and RTs data were analysed using mixed logistic regression models (Jaeger, 2008) and linear mixed effects regression models (Baayen et al., 2008), respectively. Follow-up regression analyses were used to assess the significant interaction effect by examining each level of the combinations of the related variables (Gollan and Goldrick, 2016). Sum coding was conducted when the fixed predictors in the model were categorical variables, such as Language. For instance, the categorical variable Language was contrast-coded by assigning −0.5 for Chinese, and + 0.5 for Uyghur (Schad et al., 2020). Through sum coding, the categorical variables were centred, and the main effect of each variable was properly tested. As the continuous variable Language Dominance was composed of standardised *z*-scores centred at 0, no further coding treatment was performed. Significance was evaluated by model comparisons. The chi-square statistics from the Type III sum of squares analysis were an indication of significance (Zahn, 2010). All statistical analyses were conducted in version 3.6.3 of the software R (R Core Team, 2020) with the packages of versions 1.1–21 lme4 (Bates et al., 2015) and 3.1–1 lmerTest (Kuznetsova et al., 2017).

### Lexical Decision Task

The individual data for accuracy rates (descriptive statistics shown in Table 2) were analysed using a logistic regression model. The two categorical predictors Word Type and Language, and the continuous variable Language Dominance were fit into the model as the fixed predictors. To properly model the random effects, the variables Subjects and Word Items were integrated into the model as intercept random effects (Barr et al., 2013). Concerning the sum coding for the two categorical variables Word Type and Language, the value assignment for Word Type was −0.5 for non-word and + 0.5 for word; for Language, it was −0.5 for Chinese and + 0.5 for Uyghur.

**Table 2:** Mean accuracy rates in percentage (%), mean response times (ms) of correct trials and the 95% confidence intervals (CI) from the lower bond to the upper bond for the lexical decision task by Word Type and Language.

	Accuracy rates		Response times	
	Mean	95% CI	Mean	95% CI
Non-word Uyghur	96.42	95.21–97.33	872	854–890
Word Uyghur	97.02	95.98–97.79	854	836–872
Non-word Chinese	98.71	98.13–99.11	780	762–798
Word Chinese	99.36	99.02–99.58	769	751–787

**Figure 1:**
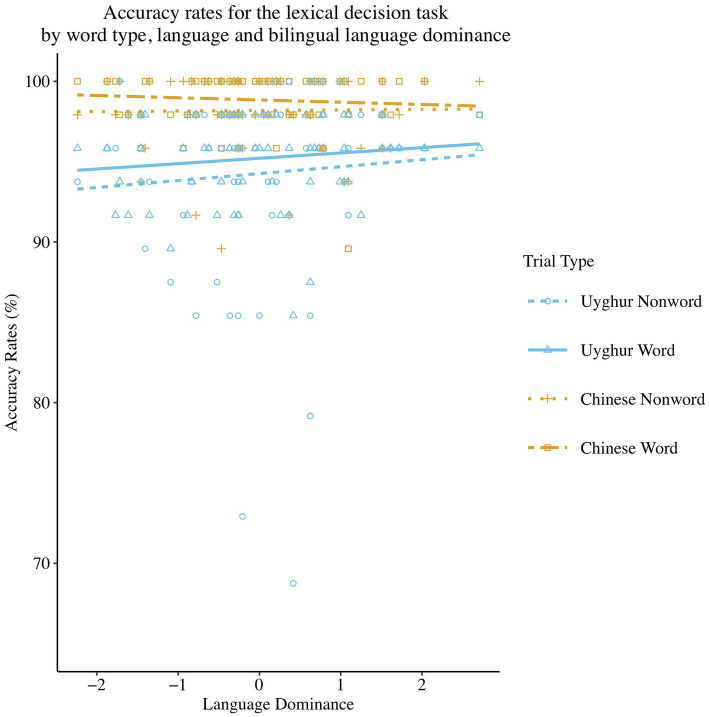
Scatterplot and regression fit lines demonstrating the relationship between Language Dominance and mean accuracy at all the combinations of variables of Language and Word Type in the lexical decision task. The score on the x-axis closer to or above +1 means a higher dominance in L1, while the score closer to or below −1 means a higher dominance in L2.

A summary of the results for the accuracy logistic model is provided in Table 3. The results reveal a significant main effect of Word Type [see Figure 1; *β* = 0.45, *SE β* = 0.15, *χ*^2^ (1) = 9.17, *p* < 0.01], with less accurate responses in the non-word trials (*M* = 97.84, *95% CI* = 97.21–98.33%) than to word trials (*M* = 98.61, *95% CI* = 98.09–98.99%). A significant main effect of Language was also found [*β* = −1.30, *SE β* = 0.15, *χ*^2^ (1) = 79.55, *p* < 0.001], showing that more accurate responses were present in the L2 Chinese (*M* = 99.09, *95% CI* = 98.77–99.32%) than in the L1 Uyghur (*M* = 96.73, *95% CI* = 95.71–97.52%). Other effects were non-significant and can be found in Table 3.

**Table 3:** Results of logistic mixed effects model on accuracy data in the lexical decision task.

	Model summary			Model effect significance		
	*β*	*SE β*	*z*	*χ^2^*	df	*p*
*Fixed effects*						
(Intercept)	4.04	0.13	31.09	**966.29**	**1**	**<0.001**
Word Type	0.45	0.15	3.03	**9.17**	**1**	**<0.01**
Language	−1.30	0.15	−8.92	**79.55**	**1**	**<0.001**
Dominance	0.03	0.09	0.35	0.12	1	0.724
Word Type * Language	−0.52	0.29	−1.76	3.08	1	0.079
Word Type * Dominance	−0.08	0.13	−0.66	0.44	1	0.508
Language * Dominance	0.14	0.13	1.16	1.34	1	0.248
Word Type * Language * Dominance	0.15	0.25	0.60	0.36	1	0.550

*Language Dominance is shortened as Dominance. Significant differences are presented in bold*.

For the RT analyses, the same fixed predictors and random factors used in the models for the accuracy scores were fit into the linear regression model. The output of this RT model is summarised in Table 4. The results showed a significant main effect of Word Type (*β* = −14.94, *SE β* = 2.71, *χ*^2^ (1) = 28.64, *p* < 0.001), with bilinguals responding more slowly to non-words (*M* = 826 ms, *95% CI* = 809–843 ms) than words (*M* = 811 ms, *95% CI* = 794–829 ms). A significant main effect of Language was found (*β* = 88.07, *SE β* = 2.67, *χ*^2^ (1) = 1089.04, *p* < 0.001) as response times to the L1 Uyghur (*M* = 863 ms, *95% CI* = 845–880 ms) were longer than to the L2 Chinese (*M* = 775 ms, *95% CI* = 757–792 ms). A significant interaction was found between Word Type and Language Dominance [*β* = 7.28, *SE β* = 2.19, *χ*^2^ (1) = 11.02, *p* < 0.001]. The follow-up regression model demonstrated that as bilinguals showed the self-reported overall language dominance shift (over time) into L2, the difference in response latencies between words and non-words becomes greater for both languages [*β* = 6.82, *SE β* = 2.28, *χ*^2^ (1) = 8.95, *p* < 0.01]. Moreover, Language and Language Dominance significantly interacted [*β* = −35.44, *SE β* = 2.19, *χ*^2^ (1) = 260.78, *p* < 0.001]. The follow-up regression model showed that bilinguals recognised the Chinese language significantly faster [*β* = 18.94, *SE β* = 9.25, *χ*^2^ (1) = 4.19, *p* < 0.05] when they were more dominant in L2 Chinese, but no effect of dominance in the L1 was found on response times to the Uyghur language [*β* = −17.04, *SE β* = 11.83, *χ*^2^ (1) = 2.08, *p* = 0.150]. Other main or two-way interaction effects were non-significant and can be found in Table 4.

**Table 4:** Results of linear mixed effects regression model on response times in the lexical decision task.

	Model summary			Model effect significance		
	*β*	*SE β*	*t*	*χ^2^*	df	*p*
*Fixed effects*						
(Intercept)	818.70	8.84	92.57	**8569.63**	**1**	**<0.001**
Word Type	−14.49	2.71	−5.35	**28.64**	**1**	**<0.001**
Language	88.07	2.67	33.00	**1089.04**	**1**	**<0.001**
Dominance	1.09	8.75	0.12	0.02	1	0.901
Word Type * Language	−7.16	5.38	−1.33	1.77	1	0.183
Word Type * Dominance	7.28	2.19	3.32	**11.02**	**1**	**<0.001**
Language * Dominance	−35.44	2.19	−16.15	**260.78**	**1**	**<0.001**
Word Type * Language * Dominance	11.60	4.39	2.64	**6.99**	**1**	**<0.01**

*Language Dominance is shortened as Dominance. Significant differences are presented in bold*.

**Figure 2:**
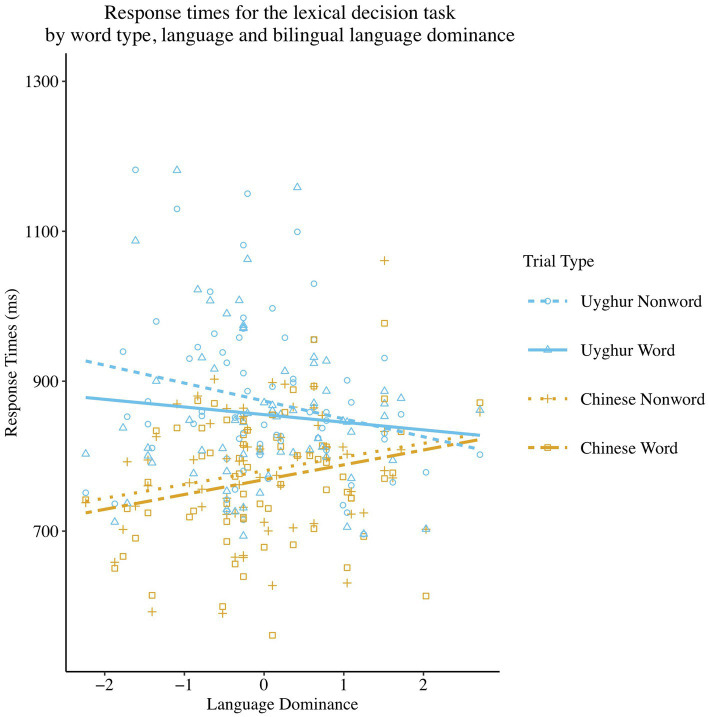
Scatterplot and regression fit lines demonstrating the relationship between Language Dominance and mean response times at all the combinations of variables of Language and Word Type in the lexical decision task. The score on the x-axis closer to or above +1 means a higher dominance in L1, while the score closer to or below −1 means a higher dominance in L2.

However, there was a significant three-way interaction (see Figure 2) between Word Type, Language and Language Dominance [*β* = 11.60, *SE β* = 4.39, *χ*^2^ (1) = 6.99, *p* < 0.01; see Table 2 for descriptive statistics]. The follow-up regression models at each level of the four combinations of Word Type and Language revealed that when bilinguals had a higher dominance in the L2, they were able to recognise Chinese words significantly faster [*β* = 19.76, *SE β* = 9.63, *χ*^2^ (1) = 4.21, *p* < 0.05] and reject Chinese non-words marginally significantly faster [*β* = 18.27, *SE β* = 9.46, *χ*^2^ (1) = 3.73, *p* = 0.053]. The rejection of Uyghur non-words [*β* = −24.34, *SE β* = 12.41, *χ*^2^ (1) = 3.84, *p* < 0.05] was significantly faster when bilinguals were more dominant in the L1, whereas no significant effect of Language Dominance was found in recognising Uyghur words [*β* = −10.50, *SE β* = 12.30, *χ*^2^ (1) = 0.73, *p* = 0.393].

### Flanker Task

The descriptive statistics for performance for the flanker task are given in Table 5. The accuracy data were analysed using a logistic model composed of the fixed predictors Stimulus Type and Language Dominance and the random factors of Subjects. For the sum coding for Stimulus Type, different sets of values were assigned to congruent trials (0.5, 0), neutral trials (−0.5, −0.5) and incongruent trials (0, 0.5). As the variable Stimulus Type was composed of three levels, pairwise comparisons were also used to demonstrate the contrasts between each level of the variable. The findings showed a significant main effect of Stimulus Type [*χ*^2^ (2) = 88.92, *p* < 0.001]. The pairwise comparisons revealed that the flanker effect was significant, with higher accuracy scores in congruent conditions (*M* = 99.72, *95% CI* = 99.46–99.86%) than in incongruent conditions (*M* = 96.71, *95% CI* = 95.84–97.41%), *β* = 2.51, *SE β* = 0.35, *z* = 7.17, *p* < 0.001, as well as that the accuracy performance in neutral trials (*M* = 99.39, *95% CI* = 99.46–99.86%) was the same as in congruent trials (*β* = 0.80, *SE β* = 0.41, *z* = 1.97, *p* = 0.119) but more accurate than in incongruent trials (*β* = −1.71, *SE β* = 0.25, *z* = −6.91, *p* < 0.001). Neither a main effect of Language Dominance nor an interaction between Stimulus Type and Language Dominance (*ps* > 0.458) was found.

**Table 5:** Mean accuracy rates in percentage (%), mean response times (ms) of correct trials and the 95% confidence intervals (CI) from the lower bond to the upper bond for the flanker task by Stimulus Type.

	Accuracy rates		Response times	
	Mean	95% CI	Mean	95% CI
Congruent	99.72	99.46–99.86	681	661–701
Neutral	99.39	99.03–99.62	667	647–687
Incongruent	96.71	95.84–97.41	746	726–766

The regression model of the RTs adopted the same fixed predictors and random factors and found a significant main effect for Stimulus Type [*χ*^2^ (2) = 1417.20, *p* < 0.001]. The pairwise comparisons showed that bilinguals responded to neutral trials (*M* = 667 ms, *95% CI* = 647–687 ms) significantly faster than congruent (*M* = 681 ms, *95% CI* = 661 – 701 ms), *β* = 13.80, *SE β* = 2.18, *t* = 6.33, *p* < 0.001 and incongruent trials (*M* = 746 ms, *95% CI* = 726–766 ms), *β* = 78.60, *SE β* = 2.22, *t* = 35.42, *p* < 0.001. A flanker effect was found in that the response speed to incongruent trials was significantly slower than to congruent trials (*β* = 64.80, *SE β* = 2.22, *t* = −29.21, *p* < 0.001). The results showed the absence of a main effect for Language Dominance and no interaction effect between these two factors of Stimulus Type and Language Dominance (*ps* > 0.144).

### Correlation Analyses

#### Correlations Among Measures of Language Recognition and Bilingual Experience

Firstly, Pearson correlation analyses were adopted to investigate whether language dominance contributed to the potential recruitment of control mechanisms during word recognition in the single-language context, and to examine the extent to which long- or short-term language experience, comprising recent language exposure (short-term) and initial age of L2 acquisition (long-term), was interrelated with language dominance and recognition ability in the single-language context. The correlation analyses were conducted between the measures of language dominance (indexed by *z*-scores), recent language exposure, the initial age of L2 acquisition and L1 and L2 word recognition ability (performance on L1 and L2 word trials). The correlation results in terms of RTs and accuracy rates are presented in Tables 6, 7. There was a significantly positive correlation between L2 word recognition and self-reported overall language dominance only in terms of RTs [*r* (68) = 0.24, *p* < 0.05]. Moreover, L2 word recognition was significantly related to age of L2 acquisition only in terms of RTs [*r* (68) = 0.34, *p* < 0.01].

**Table 6:** Bilinguals’ Pearson correlation analyses between language dominance (dominance), recent exposure, onset age of L2 acquisition (AoA L2), and language recognition performance in response times (RTs) in the lexical decision task.

	Language dominance	L1 exposure	L2 exposure	AoA L2	L1 word RTs	L2 word RTs
Language dominance	-					
L1 exposure	0.57^****^	-				
L2 exposure	−0.48^****^	−0.86^****^	-			
AoA L2	0.16	0.01	−0.03	-		
L1 word RTs	−0.10	−0.01	−0.03	−0.07	-	
L2 word RTs	0.24^*^	0.08	−0.15	0.34^**^	0.45^***^	-

*N = 70;* A larger score of language dominance indicates a greater dominance in L1.

* *p < 0.05*;

** *p < 0.01*;

*** *p < 0.001*;

**** *p < 0.0001*.

**Table 7:** Bilinguals’ Pearson correlation analyses between language dominance, recent exposure, onset age of L2 acquisition (AoA L2), and language recognition performance in accuracy rates (ACC) in the lexical decision task.

	Language dominance	L1 exposure	L2 exposure	AoA L2	L1 word ACC	L2 word ACC
Language dominance	-					
L1 exposure	0.57^****^	-				
L2 exposure	−0.48^****^	−0.86^****^	-			
AoA L2	0.16	0.01	−0.03	-		
L1 word ACC	0.12	0.17	−0.15	0.05	-	
L2 word ACC	−0.08	0.16	0.00	0.06	0.04	-

*N = 70. A larger score of language dominance indicates a greater dominance in L1*.

**** *p < 0.0001*.

#### Correlations Among Measures of Language Recognition and Cognitive Control

Secondly, further Pearson correlation analyses were conducted to explore the extent to which reliance on domain-general cognitive control occurs in the language-specific context of visual word recognition in bilinguals. Two dimensions of cognitive control—inhibitory and monitoring control abilities—were taken into consideration as potential underlying mechanisms shared with domain-specific (linguistic) control. Therefore, we examined cross-domain dependency at two levels. The first analysis was conducted to correlate the flanker effect (contrast between congruent and incongruent trials), indexing inhibitory control, with L1 or L2 word recognition (RTs or accuracy rates on word trials), L1 and L2 non-word effect (contrast between word and non-word recognition) and global performance for each single-language lexical decision task. The second analysis was conducted to correlate overall performance indexing monitoring control in the flanker task with the same language control measures as stated above. The correlation analyses mentioned above were executed for both RTs and accuracy rates. The results are shown in Table 8. In terms of RTs, it was found that all bilinguals’ better overall flanker task performance, suggested to be representing monitoring control, correlated with faster speed in L1 (*r* (68) = 0.30, *p* < 0.05) and L2 word [*r* (68) = 0.73, *p* < 0.001] recognition, and with faster speed in L1 (*r* (68) = 0.29, *p* < 0.05) and L2 global [*r* (68) = 0.76, *p* < 0.001] performance. For the accuracy analyses, L2 word recognition and L2 global performance were related to a smaller flanker effect, suggested to be representing inhibitory control [*r*_L2-word_ (68) = −0.24, *p* < 0.05; *r*_L2-global_ (68) = −0.32, *p* < 0.01]. The same L2 measures were correlated with better overall flanker task performance, suggested to be representing monitoring control [*r*_L2-word_ (68) = 0.37, *p* < 0.01; *r*_L2-global_ (68) = 0.44, *p* < 0.001].

**Table 8:** All bilinguals’ Pearson correlations between language control measured by the lexical decision task (LDT) and cognitive control measured by the flanker task at the dimension of response times (RTs) and accuracy rates (ACC).

Language control	Cognitive control	Coefficients for RTs (*N* = 70)	Coefficients for ACC (*N* = 70)
L1 word	Flanker effect	−0.06	−0.08
	Flanker monitoring	0.30^*^	0.06
L2 word	Flanker effect	0.02	−0.24^*^
	Flanker monitoring	0.73^****^	0.37^**^
L1 non-word effect	Flanker effect	0.01	0.10
	Flanker monitoring	−0.04	−0.02
L2 non-word effect	Flanker effect	0.12	0.04
	Flanker monitoring	−0.01	0.01
L1 global LDT	Flanker effect	−0.06	−0.14
	Flanker monitoring	0.29^*^	0.06
L2 global LDT	Flanker effect	0.05	−0.32^**^
	Flanker monitoring	0.76^****^	0.44^***^

* *p < 0.05*;

** *p < 0.01*;

*** *p < 0.001*;

**** *p < 0.0001*.

#### Role of Language Dominance in Relationship Between Language Recognition and Cognitive Control

Thirdly, we took a closer investigation on the role of language dominance in the correlation between domain-specific and domain-general control. Two separate correlation analyses were executed for L1- and L2-dominant bilinguals respectively, employing the same linguistic and non-linguistic measures previously adopted for all participants. Using the mean value of the language dominance *z*-score as the cutoff for dominance grouping, 33 of the participants were classified as L1-dominant bilinguals and 37 as L2-dominant bilinguals. Given that the L1-dominant (*M* = 44.73, *SD* = 5.08) and L2-dominant (*M* = 48.11, *SD* = 4.86) groups differed significantly [*t* (68) = −2.85, *p* < 0.01] from each other in the IQ measure, a partial correlation analysis was executed by controlling for IQ. The correlation results in terms of RTs and accuracy scores for each group are reported in Table 9.

**Table 9:** Pearson correlations, respectively, for L1- and L2-dominant bilinguals (controlling for IQ), between language control measured by the lexical decision task (LDT) and cognitive control measured by the flanker task at the dimension of response times (RTs) and accuracy rates (ACC).

Language control	Cognitive control	Coefficients for L2-dominant bilinguals (*N* = 37)		Coefficients for L1-dominant bilinguals (*N* = 33)	
		RTs	ACC	RTs	ACC
L1 word	Flanker effect	−0.05	0.03	−0.15	−0.17
	Flanker monitoring	0.36^*^	0.01	0.29	0.08
L2 word	Flanker effect	0.01	0.11	−0.07	−0.46^**^
	Flanker monitoring	0.69^****^	0.20	0.75^****^	0.57^***^
L1 non-word effect	Flanker effect	0.03	0.27	0.15	−0.14
	Flanker monitoring	−0.04	−0.12	0.08	0.16
L2 non-word effect	Flanker effect	0.03	0.29	0.15	−0.16
	Flanker monitoring	0.17	−0.17	−0.20	0.26
L1 global LDT	Flanker effect	−0.05	−0.23	−0.10	−0.05
	Flanker monitoring	0.36^*^	0.12	0.33	−0.03
L2 global LDT	Flanker effect	0.02	−0.20	−0.04	−0.46^**^
	Flanker monitoring	0.75^****^	0.41^*^	0.75^****^	0.52^**^

* *p < 0.05*;

** *p < 0.01*;

*** *p < 0.001*;

**** *p < 0.0001*.

In L2-dominant bilinguals, only overall flanker task performance (suggested to be representing monitoring control) correlated with a number of language measures: faster speed in L1 [*r* (34) = 0.36, *p* < 0.05] and in L2 word recognition [*r* (34) = 0.69, *p* < 0.001]; with faster speed in L1 global performance [*r* (34) = 0.36, *p* < 0.05], and with faster speed [*r* (34) = 0.75, *p* < 0.001] and higher accuracy [*r* (34) = 0.41, *p* < 0.05] in L2 global performance.

For L1-dominant bilinguals, the findings in the accuracy analyses showed that not only overall flanker task performance, suggested to be representing monitoring control [*r* (30) = 0.57, *p* < 0.001], but also the flanker effect, suggested to be representing inhibitory control [*r* (30) = −0.46, *p* < 0.01] correlated with higher accuracy in L2 word recognition. In terms of RTs analyses, L1-dominant bilinguals showed a correlation between better overall flanker task performance and faster L2 word recognition [*r* (30) = 0.75, *p* < 0.001] and L2 global performance [*r* (30) = 0.75, *p* < 0.001].

## Discussion

The focus of the present study was two-fold: first, it intended to investigate whether the variables of language dominance, onset age of L2 acquisition and recent language exposure showed an effect on the variation in word recognition in two languages in a minority–majority bilingual context. Second, it explored whether language dominance had an impact on the relationship between linguistic recognition and non-linguistic domain-general control.

### Better Performance in L2 Recognition With a Limited Role of Overall Language Dominance

The present study employed two single-language versions of the lexical decision task to assess the visual word recognition processing of Uyghur–Chinese bilinguals with varying degrees of language dominance in the minority–majority language sociolinguistic context. We found better performance in L2 than L1 in the actual lexical decision task, in which all bilinguals, irrespective of self-reported overall (or across-modality) language dominance, showed significantly better scores in L2 than in L1 word recognition, both in terms of RT and accuracy analyses. This finding of faster L2 performance in lexical decision tasks may be attributed to higher-level educational and academic experience (more written language use) in the L2 than L1 because of the higher status given to the L2 in the minority–majority language context (Goodrich and Lonigan, 2018). Our results showed that in spite of bilinguals’ self-identification as being overall more dominant in their L1, no differences were found, especially in reading dominance, and L2 scores were better in visual word recognition. This result lends support to a dynamic and language task sensitive account of language dominance, with language dominance varying for each language modality (Bahrick et al., 1994; Treffers-Daller, 2015).

We detected a striking difference in the bilinguals’ self-reported reading skill and their reading preference in relation to their L1 and L2. While bilinguals reported a higher preference for reading in the L2, they self-reported higher reading skills in the L1. When compared to their scores for the word recognition task, the participants’ self-reported preference seemed to be more in line with their actual performance than their self-reported ability. The comparison between the response times for the word recognition task and the reported difference between reading preference and reading skills may indicate that reading preference is a better indicator of actual skill than self-reported assessment. The current findings thus suggest the need to complement self-reported proficiency scores with preference ratings, especially in a sociolinguistic setting in which languages have an unequal status and a dominance shift over time might occur in the indigenous population.

Our first prediction was that higher dominance in one language might contribute to more efficient and accurate word recognition in that language, measured with the two dimensions of real word and non-word recognition. Partially consistent with this prediction, our findings showed a significantly direct relationship between the speed of L2 word recognition and self-reported L2 language dominance and a marginally significant relationship between L2 non-word and self-reported L2 language dominance. However, for word recognition in the L1, only L1 non-word rejection was associated with self-reported L1 language dominance. These findings are somewhat in line with the previous study that has demonstrated that L2 language proficiency was related to L2 word recognition performance represented by L2 switch costs in a language switching context (Bultena et al., 2015). Our results further suggest that in the recognition modality, a dominance shift over time mainly affects the process of L2 word recognition and that variations in L2 word recognition are dynamically sensitive to language dominance shift over time, without adversely affecting L1 performance.

Finer-grained analyses were also performed to check for individual differences among the bilinguals and their relationships to the recognition process in both languages. In line with preceding studies (e.g., Bonfieni et al., 2019; Sulpizio et al., 2020), we found an effect of the onset age of L2 acquisition on word recognition. Specifically, the findings demonstrated that an earlier onset age of L2 acquisition correlated with faster L2 word recognition, indicating that long-term exposure to the L2 may be critical to the better performance in L2 than L1 in the recognition modality. Interestingly, we also found a positive correlation between the speed of L2 and L1 word recognition, indicating a partly shared mechanism underlying word recognition in both languages. Given that L2 word recognition was related to the age of L2 acquisition, this finding further indicated that even though a dominance shift over time into the L2 exists, L1 word recognition is not adversely affected by this phenomenon. However, the findings also showed that no similar effect of the age of L2 acquisition was detected in relation to L1 word recognition. This seems to indicate that while the onset age of L2 acquisition might account for variations in L2 word recognition, it does not show any dependency on the relationship between L1 and L2 word recognition. Hence, it can be deduced that a non-linear relationship exists between the age of L2 acquisition or recent language exposure and word recognition. Future studies should examine whether factors, such as different patterns of language use (e.g., high frequency of language switching or not), contribute to the positive interaction between L1 and L2 word recognition.

### The Role of Language Dominance in the Association Between Bilingual Word Recognition and Domain-General Language Control

We assessed the overlap between measures of linguistic and non-linguistic control by correlating performance in the single-language lexical decision task with performance in the flanker task. Our prediction was that the measures of domain-specific control (i.e., word recognition, non-word effect and global language performance) were related to the measures of domain-global control (i.e., flanker effect and overall flanker task performance). One of our present findings is consistent with the study by Gangopadhyay et al. (2019) as there were no correlations between the non-word effect (indexed by the contrast between words and non-words) and domain-general control. Specifically, the non-word effect was neither correlated with inhibitory control, indexed by the flanker effect, nor with monitoring control, indexed by overall performance in the flanker task. To some extent, this result implies that engaging in the lexical processing of rejecting within-language lexical competitors may be a domain-specific process for bilinguals. This may suggest that the process of rejecting non-words is encapsulated within the language system (Paap et al., 2019).

However, our prediction, enlightened by prior studies (Costa et al., 2009; Struys et al., 2019), was confirmed in that monitoring control seemed to be an underlying process of L1 and L2 word recognition in visual lexical processing. In line with previous findings (Struys et al., 2019), our results regarding RTs showed that for all bilinguals, word recognition measured by L1 and L2 response latencies in word trials and global performance in each single-language lexical decision task was correlated with cognitive monitoring control, measured by overall performance in the flanker task. This suggests that bilinguals with efficient overall performance in the flanker task demonstrate faster performance in word recognition. This relationship supports the prior finding that dependency on domain-general control can be manifested when a linguistic task and non-linguistic cognitive task are structurally matched (De Baene et al., 2015) in the sense that both tasks feature an equal proportion of easy (word or congruent trials) and difficult (non-word or conflict trials) conditions, presented in an unpredictable order.

Partially consistent with our prediction that inhibitory control can be involved in both L1 and L2 word recognition, the present findings regarding accuracy rates showed that all bilinguals demonstrated a link between inhibitory control, indexed by the flanker effect, and L2 word recognition, indexed by L2 word trials and global L2 performance. This result is consistent with studies that have focused on domain generality in the bilingual recognition process (Blumenfeld and Marian, 2011; Blumenfeld et al., 2016) and reported that the recruitment of inhibitory control underlies L2 access in the process of auditory word recognition. It also lends support to the BIA model (Grainger and Dijkstra, 1992; van Heuven et al., 1998), which proposes the recruitment of top-down domain-general inhibitory control in word recognition processing. However, since inhibitory control is selectively present in L2 word recognition but not in L1 word recognition, this may further indicate that inhibitory control is sensitive to the relative strength of each language, with control being especially necessary in the language acquired later, irrespective of its current dominance. Moreover, our findings may support the notion that the minority–majority bilingual context entails an application of the coupled control mode (Green and Wei, 2014; Bosma and Blom, 2019) in which the majority L2 context seldom allows lexical insertion from the L1 minority language and thus requires inhibitory control to suppress interference from the minority L1. Such training in the sociolinguistic experience probably increases the engagement of inhibitory control.

To further examine the effect of language dominance on cross-domain overlap, follow-up separate examinations using language dominance as a categorical variable were conducted for L1- and L2-dominant bilinguals to check whether the two dominance groups differed in terms of interaction between linguistic and cognitive control. Consistent with our prediction, the correlation analyses in the dimension of accuracy showed that the degree and nature of cross-domain generality depended on language dominance. Specifically, we found that a dual mechanism of inhibitory, indexed by the flanker effect, and monitoring control, indexed by overall flanker performance, underlies L2 word recognition for L1-dominant bilinguals, whereas L2-dominant bilinguals only relied on monitoring control for L2 word recognition. That is, inhibitory control was exclusively recruited by L1-dominant bilinguals to prevent the interference of their globally dominant language (L1) during L2 performance in the recognition modality. This finding about the selective presence of inhibitory control in L1-dominant bilinguals for L2 word recognition is consistent with prior studies involving a language switching context (e.g., Mosca and de Bot, 2017), showing that inhibitory control, indexed by switch costs, was only present when bilinguals were involved with L2 word recognition before language switching, due to the previous L2 word recognition context incurring inhibitory control to constrain the dominant L1 from enhancing activation of the L2.

Our findings also suggest that once L2 proficiency has globally achieved a high level (or a dominance shift over time into L2 exists across all language skills), bilinguals no longer employ inhibitory control to facilitate the accuracy of L2 word recognition. Instead, monitoring control becomes the exclusive domain-general mechanism for L2-dominant bilinguals to process both L1 and L2 word recognition. The reason for this may be that compared to L1-dominant bilinguals, who globally maintain language strength in their native L1, L2-dominant bilinguals may increasingly enhance their L2 proficiency and thus experience a more demanding context in which their monitoring control is consistently recruited to manage the two comparably activated language systems. This finding is consistent with prior studies (Costa et al., 2009; Singh and Mishra, 2013; Chan et al., 2020), which have suggested that bilinguals with dominance in their L2 benefit from monitoring control. Our study contributes to the monitoring account that when bilinguals show a dominance shift over time into their L2, a tendency towards the exclusive involvement of the monitoring control in the word recognition process occurs.

Importantly, our results in the dimension of RTs further showed that for both the L1- and L2-dominant bilingual groups, their better overall flanker performance in the non-linguistic flanker task was correlated with more efficiency in L2 word recognition or global L2 performance in the lexical decision task. This finding suggests that in the specific context of better performance in L2 than L1 in the modality of recognition, monitoring control is crucial to L2 word recognition and might fulfil a faciliatory role in gaining access to the L2, both for those who are globally non-dominant in the L2 (L1-dominant bilinguals) and for those who have the L2 as a globally dominant language (L2-dominant bilinguals).

By taking a comprehensive view, our results suggest that monitoring is shared across L1- and L2-dominant bilinguals for both RTs and accuracy but that inhibitory control only contributes to accurate L2 word recognition in L1-dominant bilinguals. Our findings contribute to the idea that the efficiency of proactively executing domain-general monitoring control in the linguistic task is non-selective to language dominance in the context of a general dominance shift over time due to the sociolinguistic setting to which the bilinguals were exposed. Inhibitory control, in contrast, seems to be relevant only for bilinguals who are in the process of undergoing this dominance shift over time but who have not yet completed it across all language modalities. Our findings point to a dominance-based domain-general contribution to word recognition: while inhibitory control may facilitate word recognition in a language in which the bilingual has relatively low proficiency at the onset stages of second language acquisition, monitoring control may become a more important facilitator when proficiency increases. Once dominance shifts over time, monitoring control may remain relevant for word recognition in a language acquired later (even though performance is better), while the contribution of inhibitory control is reduced or even disappears. This phenomenon may occur because monitoring is particularly important when two languages are more or less balanced in strength and when control can be applied proactively through experience, while inhibitory control is particularly needed when two languages differ substantially in strength and when control is not yet highly practiced and requires reactive application.

We would like to emphasise that our findings should be assessed in light of this study’s limitations. One of limitations in our current study is that the flanker task is selected as the sole measure of domain-general control. This could be overcome in future studies by adding multiple measures of domain-general control, not only including measures of interference control as tapped into by the flanker task, but also looking at other inhibition-related tasks, such as (non-verbal variants of) the Stroop task, and the Simon task. Another limitation is that the measure of overall performance across trial type in the flanker task may not be a pure index of monitoring, because the time taken to monitor for conflicts may be embedded within stages involved with encoding, response selection and response execution. A third limitation in the current study is that only self-reported scores on the Uyghur and Chinese language were adopted for the measures of language dominance. In future studies, direct measures derived from language tests should be additionally exploited to reflect overall language dominance. For instance, each language can be tested through a single-language verbal fluency task as a measure of productive vocabulary abilities and the Peabody picture vocabulary test as a measure of receptive abilities. These two direct language tests can be used together as a composite index for evaluating language dominance. An additional limitation is related to the test order of the two language tasks (first in Chinese and then in Uyghur for all participants) and the instructions only given in Chinese. Previous research has suggested (for a review, see de Groot and Christoffels, 2006; Olson, 2017) that the language mode in which bilinguals find themselves might have an effect on the amount of control that is required on the non-target language. Features, such as test order or language of instruction, could have an impact on this language mode. We recommend, therefore, future studies that would like to replicate this study, to minimise the effect of language mode by counterbalancing not only the order of the tasks but also the languages used in instructions.

## Conclusion

Focusing on bilinguals in a minority–majority language sociolinguistic context, the current study investigated the role of bilingual dominance along with linguistic (onset age of L2 acquisition) and sociolinguistic experience (recent language exposure) in the language recognition process. It also explored the effect of language dominance on the link between language recognition (domain-specific) control and cognitive (domain-general) control. We found better performance for the majority L2 in visual lexical access in the single-language context for all bilinguals. Our findings revealed better performance in L2 than L1 in visual word recognition and suggest that the initial age of L2 acquisition (but not recent language exposure) and across-modality language dominance as a continuous variable contribute to variation in L2 recognition. Our results also support a monitoring account for bilingual language recognition in the L2, independent of language dominance. Importantly, language dominance as a categorical variable was found to play a role in across-domain generality as L2-dominant bilinguals had an exclusive reliance on domain-general monitoring control, while L1-dominant bilinguals drew on both inhibitory and monitoring control to process the later-acquired L2 in the recognition modality. Our study indicates that language dominance, operationalised as a continuous and categorical variable, shows its effect not only directly in the lexical recognition process but also indirectly as an impact on the domain-general contribution to recognition, depending on whether the bilingual reported overall dominance in their L1 or L2.

## Data Availability Statement

The raw data supporting the conclusions of this article will be made available by the authors, without undue reservation.

## Ethics Statement

The studies involving human participants were reviewed and approved by the Academic Committee of Shaanxi Normal University. The patients/participants provided their written informed consent to participate in this study.

## Author Contributions

RW contributed to the conception of the study, design of the experiments, collection and analysis of data, wrote the manuscript, and revised the manuscript critically for important intellectual content. ES advised on the conception and design of the study and provided critical revisions on the manuscript for important intellectual content. All authors contributed to the article and approved the submitted version.

## Funding

The research presented here was funded by the China Scholarship Council (CSC). Grant/Award Number: 201507650007.

## Conflict of Interest

The authors declare that the research was conducted in the absence of any commercial or financial relationships that could be construed as a potential conflict of interest.

## Publisher’s Note

All claims expressed in this article are solely those of the authors and do not necessarily represent those of their affiliated organizations, or those of the publisher, the editors and the reviewers. Any product that may be evaluated in this article, or claim that may be made by its manufacturer, is not guaranteed or endorsed by the publisher.
