# Supplementary material for: A Domain-General Monitoring Account of Bilingual Language Control in Recognition: The Role of Language Dominance and Bilingual Experience
Source: Front Psychol. 2022 Apr 12;13:854898. doi: 10.3389/fpsyg.2022.854898 (PMC9039186; doi:10.3389/fpsyg.2022.854898)

Appendix 1

48 Uyghur words

ناخشا مەكتەپ ئوغۇل رەسىم گۈزەل ياخشى يالغان كېرەك كىچىك ئاسمان

سۆزلە بىرگە تاماق سەپەر ئۈزۈن ئادەم دۇنيا پەرىق ۋاقىت دۆلەت

يالغۇز خەۋەر ئىسسىق ۋەدە مىنۇت زۆرۈر دوست كىتاب يىراق چۈنكى

ھايۋان قاتتىق -كۆر قانداق يېڭى سوۋغات نومۇر كىرىم ئائىلە پىكىر

نەرسە بۇيان باشقا قېرى ھەپتە شىركەت ئۈمۈت پىلان

Appendix 1 (continued)

48 Uyghur non-words

شفپرگئا گفئەڭ ئەتاژچ هكئەق دمگئاخ مپدئوگ مچقكجژ ژڅدلزئۇ ئەجفەپ مقچلئەۋ

قئامرس ائەزدل ئەئوكرف رگژجئو غئوئاهن بتشئو بغڭئاد خرانئۇز زدەشھ كنژئار

زەپتل چغكەقا گدنئە لكەئاچز ئەكمل رپچبكۇ رەتفجك سئوخپگ ژغخفئۇ سخزوچ

شخڭوئۆ مقئېچۇ ئىخنھۆ الگبغئۇ لخبتئائۆ زسئولق نپغاش منھاشۇ زھستئا ئاغبھگن

ئاسقژ ھلئىژنغ ڭخۆنسو جتئېژە فڭزبتە تسردئا جۇئاتبژ قچرئىۆ

Appendix 1 (continued)

48 Chinese words

时	让	多	发	听	快	动	欢	东	本
加	何	更	利	外	边	刚	每	必	医
并	早	向	决	约	字	目	报	远	布
护	光	礼	代	岁	式	划	极	步	半
冷	存	乱	呆	私	石	免	曲		

Appendix 1 (continued)

48 Chinese non-words

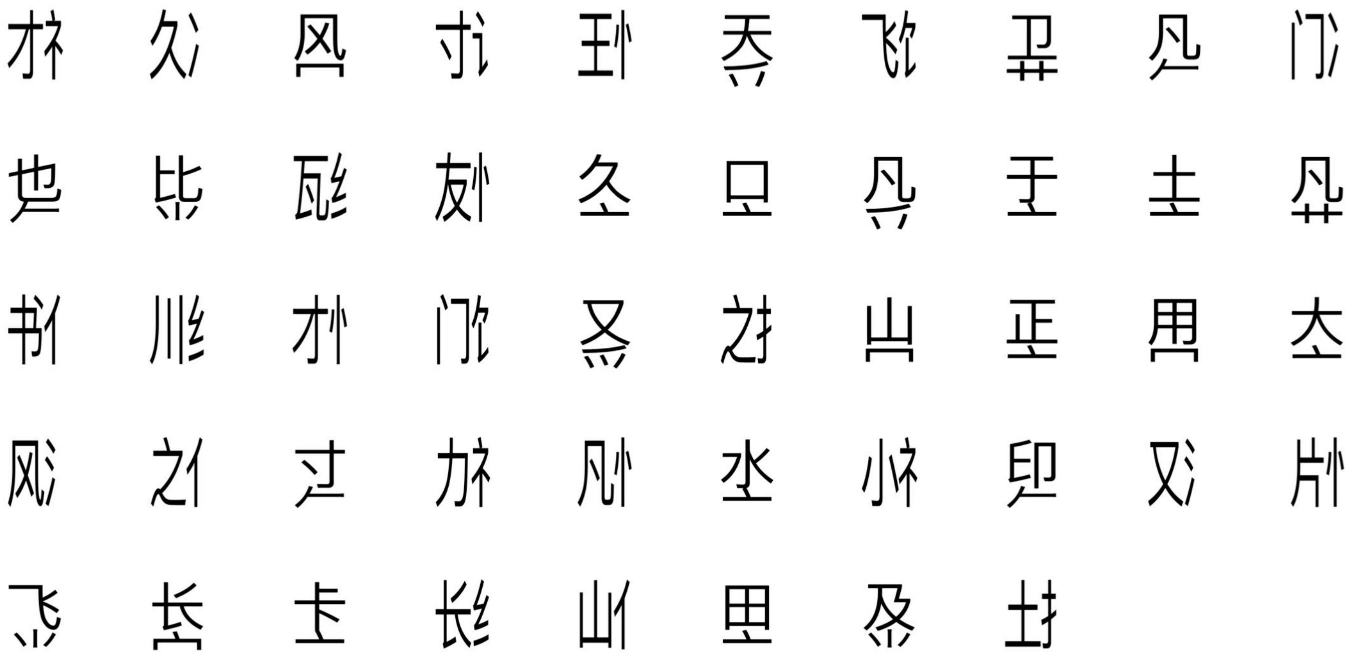

Supplement: Supplementary file 1 [file Data_Sheet_1.PDF]
